# Halloysite Nanotubes as Bimodal Lewis/Brønsted Acid Heterogeneous Catalysts for the Synthesis of Heterocyclic Compounds

**DOI:** 10.3390/nano13030394

**Published:** 2023-01-18

**Authors:** Jiaying Yu, Javier Mateos, Mauro Carraro

**Affiliations:** 1 Department of Chemical Sciences, University of Padova, Via F. Marzolo 1, 35131 Padova, Italy; 2 College of Chemistry and Environmental Engineering, Shenzhen University, 3688 Nanhai Ave, Shenzhen 518060, China; 3 ITM-CNR, UoS of Padova, Via F. Marzolo 1, 35131 Padova, Italy

**Keywords:** halloysites nanotubes, solid acids, heterogeneous catalysts, multicomponent reactions, Biginelli reaction, 3,4-dihydropyrimidinones

## Abstract

Halloysite nanotubes can be used for the preparation of solid catalysts. Owing to their natural availability at low-cost as well as to their large and easy-to-functionalize surface, they can be conveniently activated with mineral acids or derivatized with acidic groups. Nevertheless, the use of HNTs as catalysts in complex transformations is still limited. Herein, we report two strategies to utilize HNT-based materials as solid acidic catalysts for the Biginelli reaction. To this aim, two methods for increasing the number of acidic sites on the HNTs were explored: (i) the treatment with piranha solution (Pir-HNTs) and (ii) the functionalization with phenylboronic acid (in particular with benzene-1,4-diboronic acid: the sample is denoted as HNT-BOA). Interestingly, both strategies enhance the performance of the multicomponent reaction. Pir-HNTs and HNT-BOA show an increased reactivity (72% and 89% yield, respectively) in comparison with pristine HNTs (52%). Additionally, Pir-HNTs can be reused up to five times without significant performance loss. Moreover, the method also displays good reaction scope, as demonstrated by the preparation of 12 different 3,4-dihydropyrimidinones in up to 71% yield. Therefore, the described strategies are promising for enhancing the acidity of the HNTs as catalysts for the organic reaction.

## 1. Introduction

Acid catalysts play a crucial role in the activation of organic molecules, thus significantly promoting various important organic reactions [1]. Although mineral acids (i.e., H_2_SO_4_ and HCl) demonstrate high activity, they suffer from various shortcomings, such as reactor corrosion and poor catalyst separation and reuse. To this end, solid acidic catalysts have been designed to replace the mineral acids, with the multiple advantages of easier product separation and good catalysts recyclability [2,3,4]. However, the synthesis of an efficient solid acid with desirable catalytic performance, facile preparation, high environmental compatibility, as well as high thermal or chemical stability remains challenging [5].

Halloysite is a naturally available, two-layered aluminosilicate similar to kaolin, whereby the packing disorder generated by the neighboring alumina and silica layers forces them to curve and roll up, thus forming stable multi-layered tubes [6]. Halloysite nanotubes (HNTs) have been widely utilized as scaffolds for supporting metal or metal oxide nanoparticles (NPs) due to their low-cost, reduced environmental toxicity, and surface hydroxyl groups available for functionalization [7,8,9]. Nowadays, a limited number of literature reports describe the use of HNTs as solid acidic catalysts [10]. The common strategies to enable their catalytic use are (i) the conversion of the hydroxyl moieties into acidic groups or (ii) the treatment with an acidic solution. However, we think that extensive efforts are still needed to further enhance the performance of HNT-based acidic catalysts and extend their applications [11,12]. To the best of our knowledge, acidic HNT have only been applied to selective Prins cyclizations [13,14,15] and to the synthesis of naphthopyranopyrimidine derivatives through a three-component reaction between β-naphthol, aldehydes, and N,N-dimethylbarbituric acid [16,17].

Multicomponent reactions (MCRs) are reactions integrating more than two starting reagents into one single product. Due to the multiple advantages of atom economy, simplicity, convergence, and flexibility, MCRs are elegant alternatives for substituting multistep synthetic processes [18]. Moreover, such reactions provide a fascinating strategy to construct various heterocycles with meticulously designed features. MCRs include the Passerini, Ugi, Hantzsch, and Biginelli reactions, among many others [19]. Specifically, the Biginelli reaction has attracted enormous attention due to the essential therapeutic and pharmacological properties of its dihydropyrimidinone (DHPM) products [20]. DHPMs are not only medical synthons with excellent biological activities (e.g., for their antitumor, antibacterial, antiviral, and anti-inflammatory properties), but these types of compounds are also important natural products [21,22].

In general, acidic catalysts are required to improve the efficiency of the Biginelli reaction. Recently, some common Brønsted acids (such as H_2_SO_4_ and HCl) or Lewis acids (i.e., LiClO_4_, BiCl_3_, FeCl_3_, LaCl_3_, Mn(OAc)_3_, Cu(OTf)_2_) [23,24] were employed as efficient catalysts for this transformation. However, all these inorganic acids and transition metal salts suffered from issues of being corrosive, toxic, and/or difficult to separate/reuse. Although some heterogeneous catalysts (i.e., metal–organic frameworks (MOFs) [25,26,27,28], covalent–organic frameworks (COFs) [29], CoFe_2_O_4_@SiO_2_-NH_2_-Co^II^ [30]) were also applied for this MCR, their syntheses usually require multistep processes and tedious procedures. Therefore, it is highly desirable to develop other facile strategies for the synthesis of heterogeneous acid catalysts for the Biginelli reaction.

**Scheme 1:**
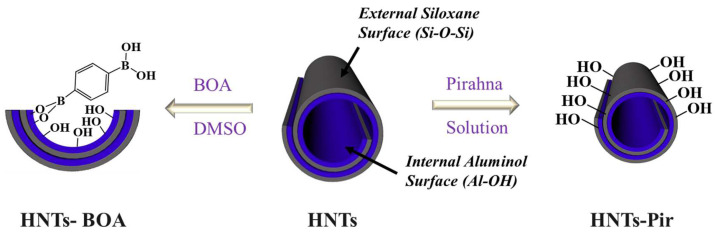
Modification of HNTs following two different approaches: (right) Pir-HNTs obtained by treatment with piranha solution, and (left) HNT-BOA obtained by grafting the diboronic acid inside the HNTs lumen.

Herein, we show two strategies for increasing the amount of acidic sites on the HNTs, aiming at improving the performance of HNTs for the Biginelli reaction. On the one hand, we treated HNTs with piranha solution to remove the impurities and activate the surface of HNTs by exposing more acidic sites on the outer silica layer [31]. The NH_3_-temperature programmed desorption (NH_3_-TPD) results indicated that the treatment with piranha solution increases the amount of Lewis acid sites on the HNTs. On the other hand, we grafted a boronic acid (BOA) on the inner lumen surface of HNTs to introduce additional acidic sites, since boronic acids are widely used and soluble in Lewis/Brønsted acid catalysts for organic reactions [32,33,34]. A diboronic acid was, thus, firmly immobilized, preferably on the internal wall of the HNTs (Scheme 1), exploiting one -B(OH)_2_ functionality for grafting the alumina while retaining the availability of the other for acidic catalysis. The resulting nano-hybrid can be easily separated from the reaction mixture for reuse.

The performances of obtained HNT-based acid catalysts prepared by the above-mentioned strategies were then demonstrated for the Biginelli reaction, showing that the HNTs with boronic acid displayed slightly higher activity than the HNTs treated with the piranha solution.

## 2. Materials and Methods

### 2.1. Reagents

Halloysite nanotubes (HNTs), anhydrous toluene, acetonitrile, ethanol, dichloromethane, dimethyl sulfoxide (DMSO), sulfuric acid (95–98 wt%), hydrogen peroxide (30 wt%), benzene-1,4-diboronic acid (BOA), ethyl acetoacetate, urea, thiourea, 3-chlorobenzaldehyde (97%), 4-chlorobenzaldehyde (97%), 2-chlorobenzaldehyde (99%), p-tolualdehyde (97%), and p-anisaldehyde (98%) were all purchased from Sigma-Aldrich.

### 2.2. Characterizations

NMR spectra were recorded on Bruker 400 Avance III HD equipped with a BBI-z grad probe head 5 mm.

High-Resolution Mass Spectra (HRMS) were collected using a Waters GCT gas chromatograph coupled with an electron ionization time-of-flight mass spectrometer (GC/MS-TOF).

FTIR spectra were obtained with solid samples on a Nicolet 5700 FT-IR instrument in ATR mode.

NH_3_-temperature programmed desorption (NH_3_-TPD) measurements were carried out on a TPDRO-1100-Omnistar with a thermal conductivity detector (TCD).

The in situ FTIR spectra during desorption of pyridine were measured on a Thermo Nicolet 380 FTIR spectrometer.

Thermogravimetric analyses (TGA) of samples were performed on a Q5000 IR instrument (TA Instruments) and collected in N2 or air upon equilibration at 100 °C, with a subsequent temperature ramp (10 °C/min) up to 1000 °C.

### 2.3. Procedures

- Grafting of boronic acid inside the lumen of HNTs

200 mg pristine HNTs and 200 mg benzene-1,4-diboronic acid (BOA) were added to 20 mL of anhydrous dimethyl sulfoxide (DMSO) under a nitrogen atmosphere, and the resulting suspension was sonicated for 30 min. After sonication, the mixture was heated at 90 °C under reflux, for 24 h, under stirring. Then, the solid phase was separated by centrifugation and washed with ethyl acetate, methanol and dichloromethane, sequentially. In the end, the prepared HNTs were kept for 24 h under a vacuum for drying. The functionalized nanotubes were labeled as HNT-BOA and characterized by FTIR and TGA analyses [35].

- Activation treatment of HNT_S_

The activation procedure was already explained elsewhere [31]. The activated HNTs were denoted as Pir-HNTs and were additionally investigated by NH_3_-TPD.

- General procedure for the synthesis of 3,4-dihydropyrimidinones

The synthesis of the 3,4-dihyropyrimidinones followed a procedure described in literation with minor changes [18]. The reaction was performed in a 25 mL round bottom flask under an N_2_ atmosphere. The substrates were added with a 1:1:1.5 stoichiometric ratio of ethyl acetoacetate (2 mmol), aldehyde (2 mmol), and urea (3 mmol) in 10 mL of acetonitrile. After the addition of 150 mg of HNTs, the mixture was kept under stirring under reflux for 38 h. After that, the catalysts were separated by filtration without waiting for the reaction mixture to cool down and washed with 20 mL of warm ethanol to remove the adsorbed reagents and products. The filtrated solution, together with 50 mL of washing ethanol, was transferred into crushed ice. As the ice melted, products appeared as white precipitates. Then, the products were purified by ethanol washing, dried under vacuum, and weighed to calculate the isolated yield. All obtained products were characterized by ^1^H and ^13^C NMR and HRMS. Meantime, the separated catalysts were washed with water and ethanol, and dried in a desiccator, ready for the recycling tests.

## 3. Results and Discussion

### 3.1. Synthesis of Halloysite Nanotube-Based Acid Catalysts

Firstly, we prepared two HNT-based acid catalysts, as shown in Scheme 1. HNTs functionalized with boronic acid on the internal wall (i.e., HNT-BOA) were synthesized by heating the mixture of HNTs and boronic acid at 90 °C under reflux for 24 h in dimethyl sulfoxide (see experimental part). The piranha-etched HNTs (i.e., Pir-HNTs) were treated with piranha solution at 90 °C for 1 h.

**Figure 1:**
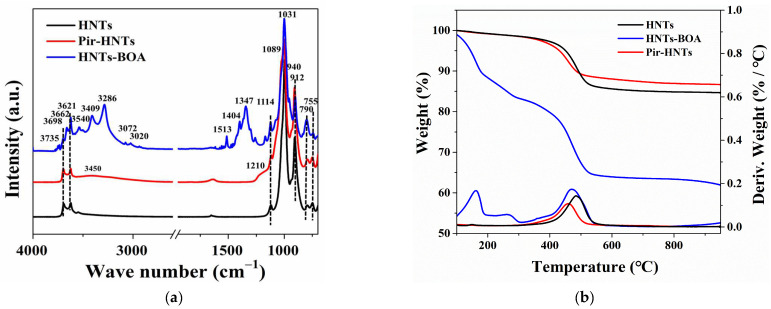
(**a**) FTIR absorbance spectra of commercial HNTs, activated HNT samples (i.e., Pir-HNTs), HNTs functionalized by boronic acid (i.e., HNT-BOA); (**b**) TGA curves of commercial HNTs, activated HNT samples (i.e., Pir-HNTs), HNTs functionalized by boronic acid (i.e., HNT-BOA).

Figure 1a displays the FTIR spectra of samples, including pristine HNTs, Pir-HNTs (i.e., after treatment of piranha solution), and HNT-BOA (i.e., functionalized with boronic acid). In the case of HNTs, the peaks situated at 3698 cm^−1^ and 3621 cm^−1^ are ascribed to the stretching vibration of Al–OH at the inner surfaces and inner interfaces (interface between the Si–O tetrahedron and Al–O octahedron) of HNTs, respectively. The bands at 940 cm^−1^ (very weak) and 912 cm^−1^ result from the deformation of Al–OH at the inner surface and inner interface, respectively [36,37]. The peaks at 1089 cm^−1^ and 1031 cm^−1^ are assigned to the in-plane Si–O stretching, while the ones at 1114 cm^−1^ and 755 cm^−1^ are related to perpendicular Si–O stretching. The peak situated at 790 cm^−1^ corresponds to the symmetric stretching of Si–O [36]. After the treatment with piranha solution, there are two additional broad signals located at 3450 cm^−1^ and 1210 cm^−1^, corresponding to Si–OH vibration and Si–O–Si stretching vibration, respectively. This observation indicates that a higher amount of hydroxyl groups is present, while amorphous silica is also produced [38].

The blue line in Figure 1 shows the spectrum of HNTs after grafting the benzene-1,4-diboronic acid (BOA). The intensity of the peak at 3698 cm^−1^ is substantially reduced after the functionalization, due to the loss of the –OH groups upon reaction with the diboronic acid. Meanwhile, the band at 3621 cm^−1^ retains its intensity, indicating that the hydroxyl groups located at the inner interfaces cannot react. The new peaks located at 3409 cm^−1^ and 3286 cm^−1^ correspond to the stretching vibration of the –OH groups linked to boron in the boronic acid [35]. The peak at 1347 cm^−1^ can be attributed to B–O stretching, confirming the presence of boronic acid in the functionalized sample HNT-BOA [39,40]. Moreover, the absorption bands at 3020 cm^−1^ and 3072 cm^−1^ are due to the C–H stretching vibration of the phenyl ring, while 1404 cm^−1^ and 1513 cm^−1^ result from the benzene skeleton vibration. Notably, all the peaks corresponding to the Si–O–Si and Si–O stretching are well kept in the spectrum of HNT-BOA, implying that the BOA is selectively grafted on the inner lumen surface of HNTs rather than the exterior surface. Besides all the bands for vibrations of halloysites and BOA, other characteristic peaks, corresponding to the small amount of solvent DMSO intercalation, can be observed: the weak peaks centered at 3735 cm^−1^ and 3662 cm^−1^ are related to hydrogen bonds generated between –OH at the inner lumen surface of HNTs and S=O groups of DMSO [41,42].

TGA measurements were used to characterize the samples and to determine the amount of BOA grafted on the HNTs (Figure 1b). The sample of HNTs after the treatment of piranha solution displays a lower overall mass loss of 12.2% than that without the treatment (15.1%) due to the removal of impurities and Al species during the treatment. As already reported, the small mass loss below 300 °C for HNTs is ascribed to the removal of adsorbed and interlayer water [37,43]. In the case of HNT-BOA, the weight loss in the temperature range of 50–300 °C is larger than that of HNTs without BOA, which may be due to the loss of intercalated DMSO and the adsorption of free BOA on the surfaces [44,45]. The mass loss in the range of 300–550 °C can be assigned to the dehydroxylation of the Al–OH and Si–OH sites of HNT in all three samples. Notably, the HNT-BOA sample displays a larger weight loss than that of HNTs without BOA; indeed, the additional weight loss can be attributed to the thermal decomposition of grafted BOA [46], calculated to be ca. 5.6 wt%.

**Figure 2:**
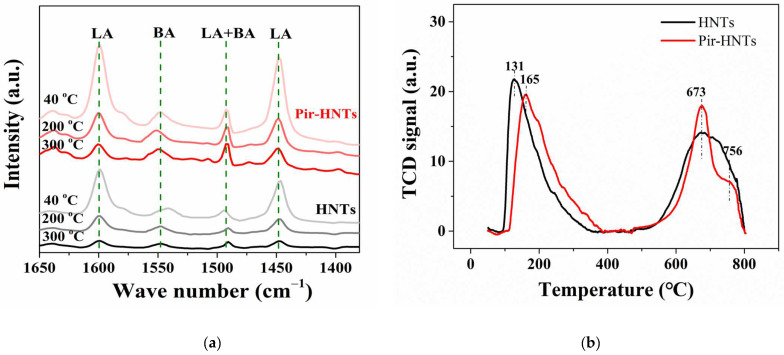
(**a**) Py-IR spectra of commercial HNT and activated HNT samples (i.e., Pir-HNTs) at different desorption temperatures: 40, 200, and 350 °C; (**b**) NH_3_-TPD profiles of commercial HNT and activated HNT samples (i.e., Pir-HNTs). TCD = thermal conductivity detector.

To investigate the nature of the acidic sites on the HNTs and pir-HNTs, IR spectroscopy of adsorbed pyridine (Py-IR) measurements was performed on these two samples (Figure 2). Both samples demonstrated various peaks corresponding to pyridine adsorbed on the Lewis acid (LA) sites (1601 and 1448 cm^−1^) and Brownsted acid (BA) sites (1548 cm^−1^) [47,48]. Moreover, the type of acidic sites for the peak at 1492 cm^−1^ cannot be distinguished by Py-IR, and such a peak is usually assigned to both LA and BA sites [40]. The amount of different kinds of acid sites was further quantified according to the peak area. As shown in Table S1, despite the nearly similar amount of BA sites on them, the amount of LA sites on the pir-HNTs is 1.84-fold higher than that of HNTs at 40 °C, suggesting that the treatment of piranha solution can significantly increase the number of LA sites on the HNTs but has little impact on that of BA sites. With the temperature increasing from 40 to 200 and 350 °C, the number of LA sites on both samples gradually decrease, due to the loss of weaker LA sites. Notably, at 350 °C, the number of LA sites on the pir-HNTs is still as high as 22.79 μmol/g corresponding to strong LA sites (SLA), 3.74-fold to that of HNTs, indicating that the piranha solution can also markedly enhance the strength of LA sites for promoting the generation of SLA sites. Interestingly, the strength of BA is also enhanced through the treatment of piranha solution, and the amount of BA for the pir-HNTs (21.57 μmol/g) is much higher than that of HNTs (9.00 μmol/g) at 350 °C. The acidic sites on the above two samples were further examined via the method of NH_3_-temperature programmed desorption (NH_3_-TPD, Figure 2b). There are two desorption peaks centered at around 150 °C and 700 °C in both cases, which correspond to BA and SLA sites on the surface of HNTs, respectively [47]. After the treatment with piranha solution, the peak of weak acid sites moves from 131 °C for the HNTs, and to 165 °C for the Pir-HNTs, suggesting that the strength of BA sites is enhanced for Pir-HNTs. Moreover, compared with the HNTs, the intensity of the peak centered at 673 °C for the Pir-HNTs is increased, indicating that the piranha solution treatment can increase the number of strong acidic sites on the HNTs. However, the amount of super acidic sites (700–800 °C) decreased after piranha solution treatment. Nevertheless, according to the Sabatier principle, the super strong acidic sites may cause excessive adsorption of molecules, which may hinder the reactivity. Thus, the decreased amount of super-strong acidic sites after the treatment may be beneficial for the multicomponent reaction [49].

Therefore, the results of Py-IR match well with those of NH_3_-TPD and undoubtedly verify that the piranha solution treatment can markedly increase the amount of LA sites, especially SLA sites, and enhance the strength of BA sites on the HNTs.

It is reported that HNTs possess both Brønsted and Lewis acid sites. The Brønsted acid sites originated from the acidic OH-groups (H_3_O^+^), while Lewis acid sites are offered by the coordinatively unsaturated Al^3+^ ions [15,50,51]. As described in the previous literature, we found that piranha treatment could remove the impurities, including Na^+^, K^+^, etc., from HNTs, slightly enlarge the lumen cavity and significantly increase the specific surface area [38]. As a result of the three effects induced by the piranha solution, the number of Al acidic sites and of Si–OH groups can both be increased.

NH_3_-TPD and Py-IR methods cannot be used to measure the acidic properties of HNT-BOA since the benzene-1,4-diboronic acid undergoes thermal decomposition, as shown in the above TGA results. Nevertheless, boronic acids are well-known acid catalysts (both Brønsted and Lewis acid) that have been used as catalysts for various organic reactions [34,52,53,54]. In the future, the acidity may be investigated by potentiometric acid titration (PT).

### 3.2. Catalytic Performance of HNTs in the Biginelli Reaction

**Scheme 2:**
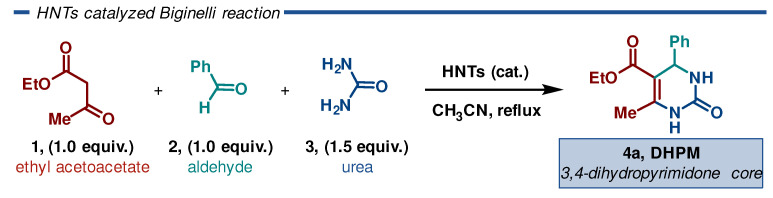
Biginelli reaction catalyzed by HNTs.

The Biginelli reaction is a well-known acid-catalyzed process, and it has drawn extensive attention from chemists due to the attractive pharmacological properties of its DHPM products accessible through this manifold. The catalytic performance of pristine and modified HNTs was evaluated using the Biginelli reaction as a model reaction for generating 3,4-dihydropyrimidinones using widely available feedstock materials. The reaction was carried out under optimized conditions: a 1:1:1.5 stoichiometric ratio of ethyl acetoacetate **1** (2 mmol), aldehyde **2** (2 mmol), and urea **3** (3 mmol) in 10 mL of acetonitrile under reflux (Scheme 2).

**Figure 3:**
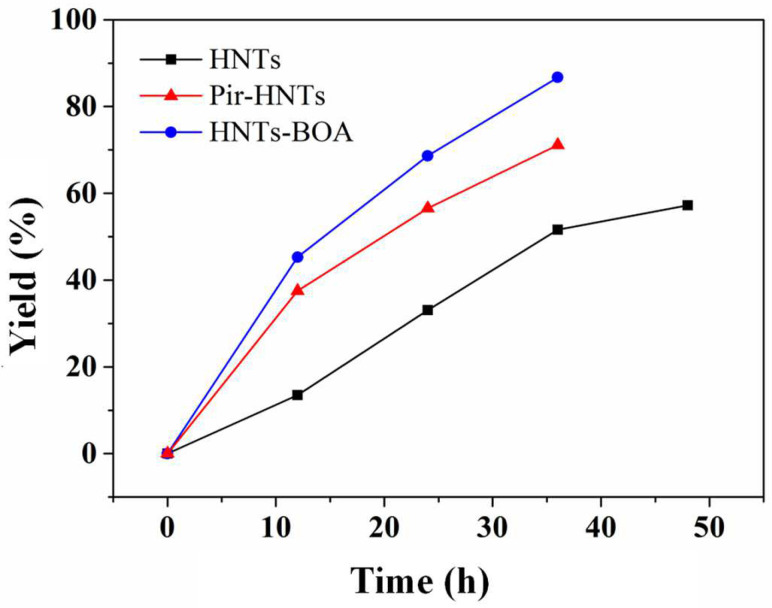
Yields of products dependent on the reaction time over different catalysts, including commercial HNTs, Brønsted-acid-activated HNTs (Pir-HNTs), and HNTs functionalized by boronic acid (HNT-BOA).

Figure 3 shows the isolated yield reaction profiles of the Biginelli reaction over time in the presence of different HNTs catalysts. The tested catalysts were: (i) commercial HNTs (black squares, Figure 3), (ii) Brønsted acid-activated HNT samples (Pir-HNTs, red triangles, Figure 3), and (iii) Lewis acid HNTs functionalized (HNT-BOA, blue circles, Figure 3).

In order to carefully determine the reaction performance over time, the yields were calculated after product isolation and purification for all the catalysts. We started our investigations by testing the activity of HNTs. It is worth mentioning that pristine HNTs exhibited moderate activity in the Biginelli reaction due to the intrinsic presence of acidic sites on it. The yields of products catalyzed by HNTs gradually increased from 13% yield after 12 h to 52% yield after 36 h. Under a longer reaction time (48 h), the yield of **4a** (57%) is only slightly improved. Interestingly, both silica and alumina, taken as reference compounds, did not allow to obtain yields higher than 10%, highlighting the importance of the two-layered aluminosilicate material as a catalyst (Table S2). Then, we decided to test the performances of the treated HNTs. In fact, the yields of **4a**, when catalyzed by piranha solution-treated HNTs, are 38% in 12 h and 72% in 36 h (three times faster than pristine HNTs). The enhanced catalytic performance of the Pir-HNTs is attributed to the increased number of acidic sites resulting from the treatment with piranha solution. Analogously, the HNTs grafted with the BOA sample exhibit the best catalytic performance among these three catalysts. In the case of utilizing HNT-BOA as a catalyst, the yields of DHPMs are 45% in 12 h and up to 87% after 36 h (almost five times faster than pristine HNTs). This enhancement by using HNT-BOA as catalysts suggests that the functionalization of HNTs with the BOA can efficiently boost the performance as acidic catalysts in MCR. To sum up, the experimental results indicated that both protocols are efficient in increasing acidity sites, leading to a higher activity of related acid-catalyzed reactions.

**Figure 4:**
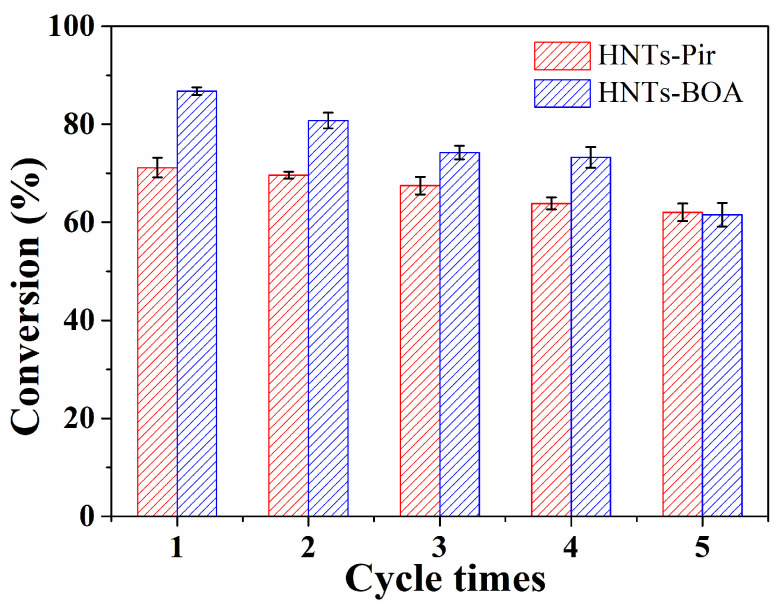
The recycling test of Pir-HNTs and HNT-BOA for Biginelli reaction to produce 3,4-dihydropyrimidinones (**4a**).

One of the most reliable tests for the versatility of a heterogeneous catalyst is by checking its recyclability. For this reason, Pir-HNTs and HNT-BOA were evaluated, as shown in Figure 4. After every reaction, the catalyst was recovered by simply filtrating the reaction mixture and washing the obtained solid with ethanol and water. Clearly, the Pir-HNTs demonstrated excellent stability for the Biginelli reaction (red bars, Figure 4) with minor activity loss during five recycles (less than 10% yield erosion). In contrast, an activity decay of up to 25% was observed with the HNT-BOA (blue bar, Figure 4) after five recycles. These results suggest a poorer stability of the HNT-BOA in this system when overused.

The reason for the decreased activity in subsequent recycles can be ascribed to the mechanical damages occurring during mixing and reaction workup. Moreover, HNT-BOA is more susceptible to the leaching of organic groups.

Given that the treatment with piranha solution is easier and amenable to scaling-up than the grafting of boronic acid and owing to their higher robustness during catalytic turnover, Pir-HNTs were selected to explore the scope of the HNTs catalyzed via Biginelli reaction (Table 1).

On the one side, diversely substituted aldehydes **2a**–**g** bearing either electron-donating or electron-withdrawing groups were tested. The functional group on the aryl moiety of the aldehydes had no significant effect on the formation of DHPM products **4a**–**h**. Electron-rich and electron-poor aldehydes achieved 3,4-dihydropyrimidones in good yields (from 46 to 71% yield). At the same time, substituting the urea **3a** with thiourea **3b** furnished **4i** in a good 62% yield. Analogously, using methyl acetoacetate **1b**, also yielded **4j** in good yields (69%), altogether showing the high versatility of HNT catalysts for MCRs.

The activity of our catalysts toward the **4a** product has been compared with that of other solid acid catalysts, collecting relevant data in Table S3 [55,56,57,58]. The performance of HNTs derivatives is generally lower than that of other reported catalysts. Nevertheless, the synthetic procedure of our catalysts is easier and performed under milder conditions. Moreover, they are derived from naturally abundant HNTs with the advantages of being cheaper and environmentally friendly.

## 4. Conclusions

In conclusion, we have demonstrated that HNTs can be employed as robust acid catalysts for the Biginelli reaction to produce heterocyclic compounds, enhancing the catalytic activity of pristine HNTs via two different treatment strategies. One approach is to expose more acidic sites of the HNTs by treatment with a piranha solution. The other strategy is to introduce additional acid sites into HNTs via the functionalization with phenylboronic acid. Both strategies were found to be efficient in increasing the performance of the Biginelli reaction. By comparing the two strategies, we found that the piranha treatment is more facile to be operated, and the catalyst displayed good recycling ability. Additionally, the HNTs functionalized with boronic acid showed even higher yields compared to the Pir-HNTs. Meanwhile, as a grafted catalyst, the HNT-BOA may also have the problem of releasing BOA during the recycling of the reaction due to the cleavage of B–O bonds. Nevertheless, since boronic acid can be considered a generally acid catalyst, the HNT-BOA may have the potential to apply for some specific reactions which may not occur with Pir-HNTs. In addition, the HNT-BOA catalyst can be regarded as a micro-reactor operating inside the lumen cavity of the HNTs, which may facilitate the reaction due to spatial confinement effects.

Overall, both strategies are efficient in enhancing the acidity of the HNTs for organic reaction and offer further possibilities according to the different requirements. Although other synthetic heterogeneous systems appear faster than the present solid acid (with conversions occurring in 0.5–2 h), we believe that the activated HNTs should deserve more attention, being cheaper and easier to prepare [17,18,19,20,21].

## Figures and Tables

**Table 1:** Versatility of the HNTs catalyzed via Biginelli reaction.

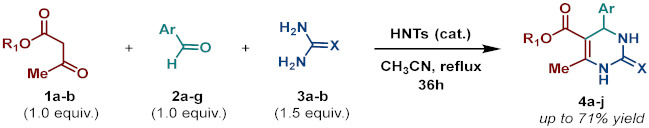 *HNTs catalyzed Biginelli reaction—Reaction scope*				
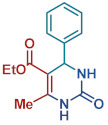	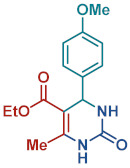	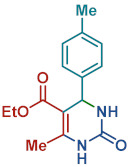	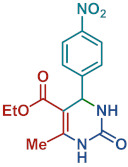	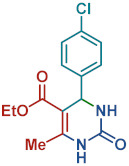
**4a** Yield 71 %	**4b** Yield 46 %	**4c** Yield 70 %	**4d** Yield 65 %	**4e** Yield 54 %
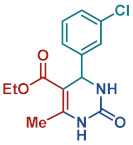	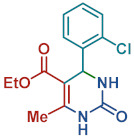	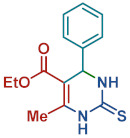	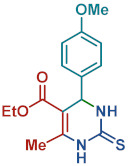	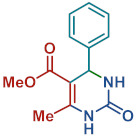
**4f** Yield 64 %	**4g** Yield 68 %	**4h** Yield 56 %	**4i** Yield 62 %	**4j** Yield 69 %

Reaction Conditions: 1 (2.0 mmol), 2 (2.0 mmol), 3 (2.0 mmol), catalysts (150 mg), 10 mL CH_3_CN under reflux temperature, 36 h. Isolated yields after recrystallization are reported.

## Data Availability

Additional information is available from the authors.

## Supplementary Materials

The following supporting information can be downloaded at: https://www.mdpi.com/article/10.3390/nano13030394/s1, Figures S1–S20: NMR spectra; Table S1: The amount different kinds of acid sites on the HNTs and pir-HNTs determined by the measurement of Py-IR; Table S2: Yields of product **4a** over different catalysts; Table S3: Comparison of HNTs activity with respect to other reported catalysts toward Biginelli reaction.
