# Supplementary material for: Halloysite Nanotubes as Bimodal Lewis/Brønsted Acid Heterogeneous Catalysts for the Synthesis of Heterocyclic Compounds
Source: Nanomaterials (Basel). 2023 Jan 18;13(3):394. doi: 10.3390/nano13030394 (PMC9919349; doi:10.3390/nano13030394)
Supplement: Supplementary file 1 [file nanomaterials-13-00394-s001.zip › nanomaterials-2157378-supplementary.pdf]

Halloysite nanotubes as bimodal Lewis/Brønsted acid heterogeneous catalysts for the synthesis of heterocyclic compounds

Jiaying Yu, Javier Mateos, Mauro Carraro

Experimental section

NH_3-temperature programmed desorption (NH_3-TPD)

The samples of HNTs and Pir-HNTs (200 mg) were put under a flow of He to remove adsorbed gas on the surfaces. Then the samples were treated with a flowing ammonia gas mixture (5% NH_3 in He) at 30 °C for 1.5 h. Chemisorbed NH_3 was desorbed by heating from 30°C up to 700 °C at a rate of 10 °C min^{-1}.

Pyridine

In-situ pyridine-FT-IR spectra were collected on a Thermo Nicolet 380 FT-IR spectrometer. In typical process, the surface of samples was first degassed at 200 ℃ under Ar atmosphere for 30 min, and then a background spectrum was collected after cooling down to room temperature. Subsequently, pyridine vapor was poured into the system until equilibrium was reached, followed by Ar blowing for 30 min to remove the physically adsorbed pyridine. Finally, the FT-IR spectra at different temperature (40 ℃, 200 ℃ and 300 ℃) were collect in order to determine the strength of acidic sites.

Table S1 The amount different kinds of acidic sites on the HNTs and pir-HNTs determined by the measurement of Py-IR

Samples	Temperature (ºC)	Amount of Brøsted acid sites (μmol/g)	Amount of Lewis acid sites (μmol/g)	Total amount of acidic sites (μmol/g)
HNTs	40	20.26	59.03	79.29
	200	13.80	15.08	28.88
	350	9.00	6.10	15.10
pir-HNTs	40	25.93	108.43	134.35
	200	23.46	36.73	59.83
	350	21.57	22.79	44.35

Table S2 Yields of product **4a** over different catalysts

Entry	Catalyst	Yield [%]
1	HNTs	52
2	Pir-HNTs	72
3	HNTs-BOA	87
4	Silica	<10
5	Alumina	<6

Conditions: ethyl acetoacetate 1 (2 mmol), aldehyde 2 (2 mmol) and urea 3 (3 mmol), catalysts (150 mg), 10 mL CH_3CN under reflux temperature, 36 h.

Table S3 Comparison of HNTs activity with respect to other reported catalysts towards the Biginelli reaction.

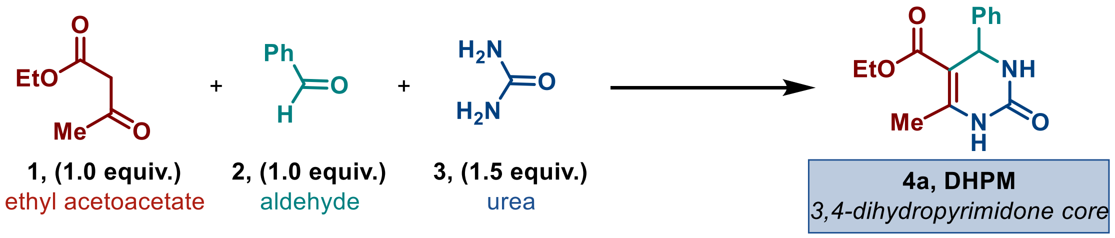

Entry	Catalysts	Yield of **4a** (%)	Time (h)	
1	HNTs	52	36	*This work*
2	Pir-HNTs	72	36	*This work*
3	HNTs-BOA	87	36	*This work*
4	TSA/MIL-88	72	4	*ACS Appl. Nano Mater*. 2022, 5, 16987
5	PS-PEG-SO_3H	86	10	*Catal. Commun*. 2009, 10, 1146
6	Carbon-SO_3H	89	4	*Terahedron Lett.* 2012, 53, 1968
7	(0.5)IL-BF_4@MCM-41	65	8	*J. Mol. Liq.* 2018, 262, 328

Characterization of the Biginelli reactions' products

NMR spectra were recorded on Bruker 400 Avance III HD equipped with a BBI-z grad probe head 5mm. The chemical shifts (δ) for ^{1}H and ^{13}C are given in ppm relative to residual signals of the solvents (DMSO-d_6 @ 2.50 ppm ^{1}H NMR, 39.52 ppm ^{13}C NMR). Coupling constants are given in Hz. The following abbreviations are used to indicate the multiplicity: s, singlet; d, doublet; t, triplet; q, quartet; m, multiplet; br, broad signal.

All obtained ***3,4-dihydropyrimidinones (DHPMs)*** were characterized by ^{1}H and ^{13}C NMR and HRMS, as follows

4a. Ethyl-1,2,3,4-tetrahydro-6-methyl-2-oxo-4-phenylpyrimidine-5-carboxylate

^{1}H NMR (400 MHz, DMSO-d_6) : δ 9.24 (s, 1H); 7.79 (s, 1H); 7.36-7.28 (m, 5H), 5.18 (d, *J* = 3.0 Hz, 1H). 4.02 (q, *J* = 6.6 Hz, 2H), 2.28 (s, 3H), 1.13 (t, *J* = 6.1 Hz, 3H)

^{13}C NMR (101 MHz, DMSO-d_6): δ 165.57, 152.54, 148.66, 145.29, 128.82, 127.94, 126.35, 99.47, 59.54, 54.14, 18.26, 14.41.

HRMS (ESI-MS) calculated for $C_{14}H_{17}N_2O_3^+$ [M+H$^+$] 261.1161, found 261.1231.

4b. Ethyl 4-(4-methoxyphenyl)-6-methyl-2-oxo-1,2,3,4-tetrahydropyrimidine-5-carboxylate

^{1}H NMR (400 MHz, DMSO-d_6): δ 9.20 (s, 1H), 7.72 (s, 1H), 7.18 (d, *J* = 8.6 Hz, 2H), 6.91 (d, *J* = 8.6 Hz, 2H), 5.12 (s, 1H), 4.01 (q, *J* = 7.1 Hz, 2H), 3.75 (s, 3H), 2.27 (s, 3H), 1.14 (t, *J* = 7.1 Hz, 3H).

^{13}C NMR (101 MHz, DMSO-d_6): δ 165.58, 158.61, 152.53, 148.58, 137.47, 127.85, 114.10, 100.13, 59.53, 55.35, 53.83, 18.03, 14.33.

HRMS (ESI-MS) calculated for $C_{15}H_{19}N_2O_4^+$ [M+H$^+$] 291.1267, found 291.1341.

4c. Ethyl 6-methyl-2-oxo-4-(*p*-tolyl)-1,2,3,4-tetrahydropyrimidine-5-carboxylate

^{1}H NMR (400 MHz, DMSO-d_6): δ 9.21 (s, 1H), 7.74 (s, 1H), 7.15 (s, 4H), 5.14 (s, 1H), 4.01 (q, *J* = 6.9 Hz, 2H), 2.28 (d, *J* = 9.0 Hz, 6H), 1.14 (t, *J* = 7.0 Hz, 3H).

^{13}C NMR (101 MHz, DMSO-d_6): δ 165.69, 152.63, 148.56, 142.54, 136.83, 129.26, 126.47, 99.90, 59.41, 54.29, 21.12, 18.06, 14.69.

HRMS (ESI-MS) calculated for $C_{15}H_{19}N_2O_3^+$ [M+H$^+$] 275.1317, found 275.1410.

4d. Ethyl 6-methyl-4-(4-nitrophenyl)-2-oxo-1,2,3,4-tetrahydropyrimidine-5-carboxylate

^{1}H NMR (400 MHz, DMSO-d$_6$): δ 9.41 (s, 1H), 8.26 (d, *J* = 8.6 Hz, 2H), 7.95 (s, 1H), 7.54 (d, *J* = 8.6 Hz, 2H), 5.31 (s, 1H), 4.02 (q, *J* = 7.0 Hz, 2H), 2.30 (s, 3H), 1.13 (t, *J* = 7.1 Hz, 3H).

^{13}C NMR (101 MHz, DMSO-d$_6$): δ 165.51, 152.45, 152.00, 149.81, 147.16, 128.12, 124.32, 98.05, 59.76, 54.06, 18.28, 14.59.

HRMS (ESI-MS) calculated for $C_{14}H_{16}N_3O_5^+$ [M+H$^+$] 306.1012, found 306.1113.

4e. Ethyl 4-(4-chlorophenyl)-6-methyl-2-oxo-1,2,3,4-tetrahydropyrimidine-5-carboxylate

^{1}H NMR (400 MHz, DMSO-d$_6$): δ 9.29 (s, 1H), 7.82 (s, 1H), 7.43 (d, *J* = 7.7 Hz, 2H), 7.28 (d, *J* = 7.8 Hz, 2H), 5.17 (s, 1H), 4.02 (q, *J* = 13.2, 6.3 Hz, 2H), 2.28 (s, 3H), 1.13 (t, *J* = 6.7 Hz, 3H).

^{13}C NMR (101 MHz, DMSO-d$_6$): δ 165.67, 152.55, 149.06, 144.26, 132.21, 128.88, 128.66, 99.26, 59.67, 53.78, 18.39, 14.55.

HRMS (ESI-MS) calculated for $C_{14}H_{16}ClN_2O_3^+$ [M+H$^+$] 295.0771, found 295.0845.

4f. Ethyl 4-(3-chlorophenyl)-6-methyl-2-oxo-1,2,3,4-tetrahydropyrimidine-5-carboxylate

^{1}H NMR (400 MHz, DMSO-d$_6$): δ 9.32 (s, 1H), 7.85 (s, 1H), 7.34 (m, 4H), 5.19 (s, 1H), 4.11 – 3.96 (m, 2H), 2.29 (s, 3H), 1.13 (t, *J* = 6.9 Hz, 3H).

^{13}C NMR (101 MHz, DMSO-d$_6$): δ 165.51, 152.31, 149.45, 147.70, 133.27, 130.89, 127.72, 126.63, 125.37, 98.92, 59.59, 53.91, 18.19, 14.14.

HRMS (ESI-MS) calculated for $C_{14}H_{16}ClN_2O_3^+$ [M+H$^+$] 295.0771, found 295.0845.

4g. Ethyl 4-(2-chlorophenyl)-6-methyl-2-oxo-1,2,3,4-tetrahydropyrimidine-5-carboxylate

^{1}H NMR (400 MHz, DMSO-d6): δ 9.32 (s, 1H), 7.76 (s, 1H), 7.44 (d, *J* = 7.7 Hz, 1H), 7.38 – 7.28 (m, 3H), 5.67 (s, 1H), 3.93 (q, *J* = 7.0 Hz, 2H), 2.34 (s, 3H), 1.03 (t, *J* = 7.1 Hz, 3H).

^{13}C NMR (101 MHz, DMSO-d$_6$): δ 165.36, 151.59, 149.57, 142.12, 132.13, 129.81, 129.54, 129.24, 128.21, 98.33, 59.43, 51.91, 31.03, 17.99, 14.28.

HRMS (ESI-MS) calculated for $C_{14}H_{16}ClN_2O_3^+$ [M+H$^+$] 295.0771, found 295.0845.

4h. Ethyl 6-methyl-4-phenyl-2-thioxo-1,2,3,4-tetrahydropyrimidine-5-carboxylate

^{1}H NMR (400 MHz, DMSO-d$_6$): δ 10.39 (s, 1H), 9.71 (s, 1H), 7.42 – 7.22 (m, 5H), 5.20 (s, 1H), 4.05 (q, *J* = 13.8, 6.8 Hz, 2H), 2.33 (s, 3H), 1.14 (t, *J* = 7.0 Hz, 3H).

^{13}C NMR (101 MHz, DMSO-d$_6$): δ 174.66, 165.54, 145.58, 143.95, 128.94, 128.15, 126.84, 101.23, 59.96, 54.37, 17.55, 14.40.

HRMS (ESI-MS) calculated for $C_{14}H_{17}N_2O_2S^+$ [M+H$^+$] 277.0932, found 277.1039.

4i. Ethyl 4-(4-methoxyphenyl)-6-methyl-2-thioxo-1,2,3,4-tetrahydropyrimidine-5-carboxylate

^{1}H NMR (400 MHz, DMSO-d$_6$): δ 10.34 (s, 1H), 9.65 (s, 1H), 7.16 (d, *J* = 8.4 Hz, 2H), 6.94 (d, *J* = 8.4 Hz, 2H), 5.14 (d, *J* = 2.9 Hz, 1H), 4.01 (s, 2H), 3.76 (s, 3H), 2.32 (s, 3H), 1.14 (t, *J* = 7.0 Hz, 3H).

^{13}C NMR (101 MHz, DMSO-d$_6$): δ 174.33, 165.61, 159.18, 145.22, 136.05, 127.82, 114.18, 101.39, 59.85, 55.56, 53.83, 17.55, 14.51

HRMS (ESI-MS) calculated for $C_{15}H_{19}N_2O_3S^+$ [M+H$^+$] 307.1038, found 307.1113.

4j. Methyl 6-methyl-2-oxo-4-phenyl-1,2,3,4-tetrahydropyrimidine-5-carboxylate

^{1}H NMR (400 MHz, DMSO-d$_6$): δ 9.22 (s, 1H), 7.75 (s, 1H), 7.33-7.24 (m, 5H), 5.14 (s, 1H), 3.54 (s, 3H), 2.25 (s, 3H).

^{13}C NMR (101 MHz, DMSO-d$_6$): δ 166.31, 152.63, 149.14, 145.13, 128.93, 127.77, 126.63, 99.47, 54.25, 51.27, 18.30.

HRMS (ESI-MS) calculated for $C_{13}H_{15}N_2O_3^+$ [M+H$^+$] 247.1004, found 247.1118.

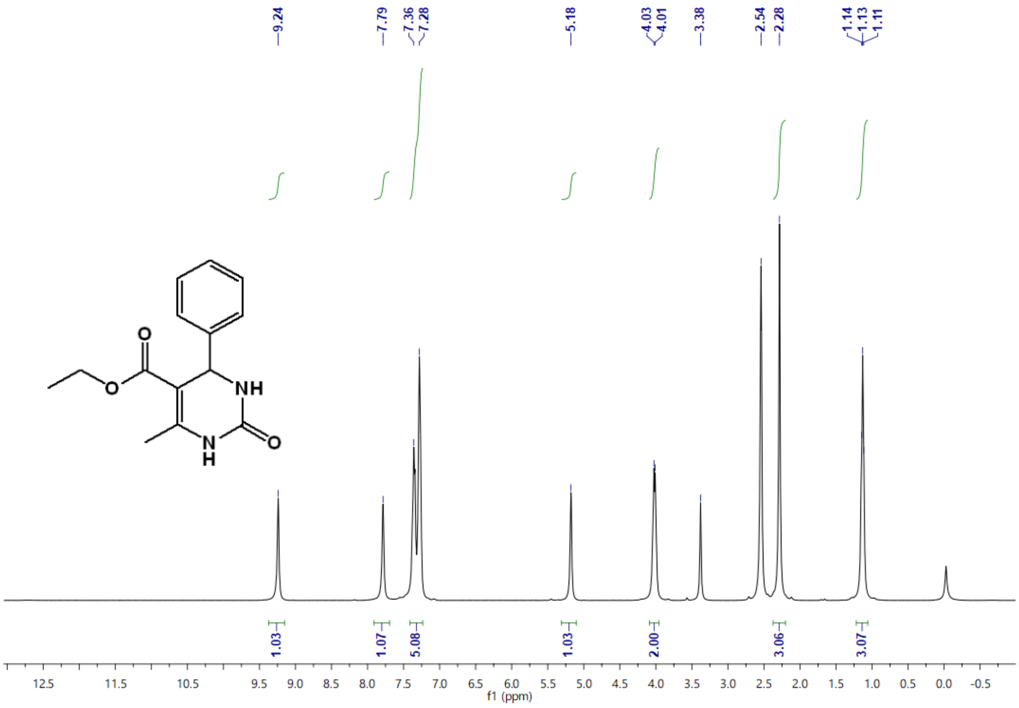

Figure S1 ^{1}H NMR **(400 MHz, DMSO-d$_6$)** spectrum of **4a**

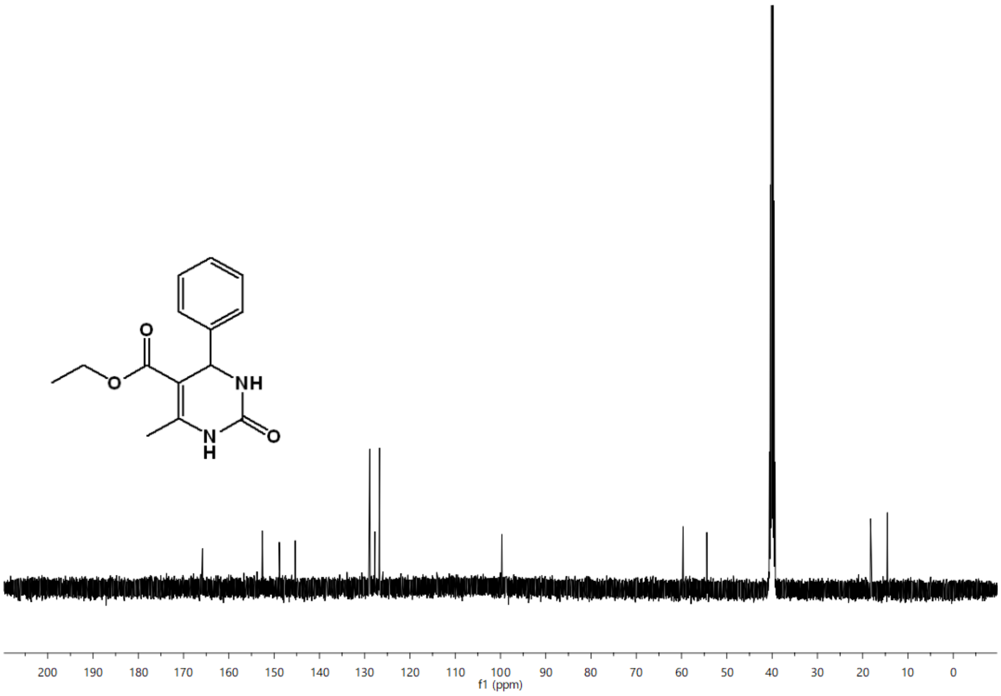

Figure S2 ^{13}C NMR **(101 MHz, DMSO-d$_6$)** spectrum of **4a**

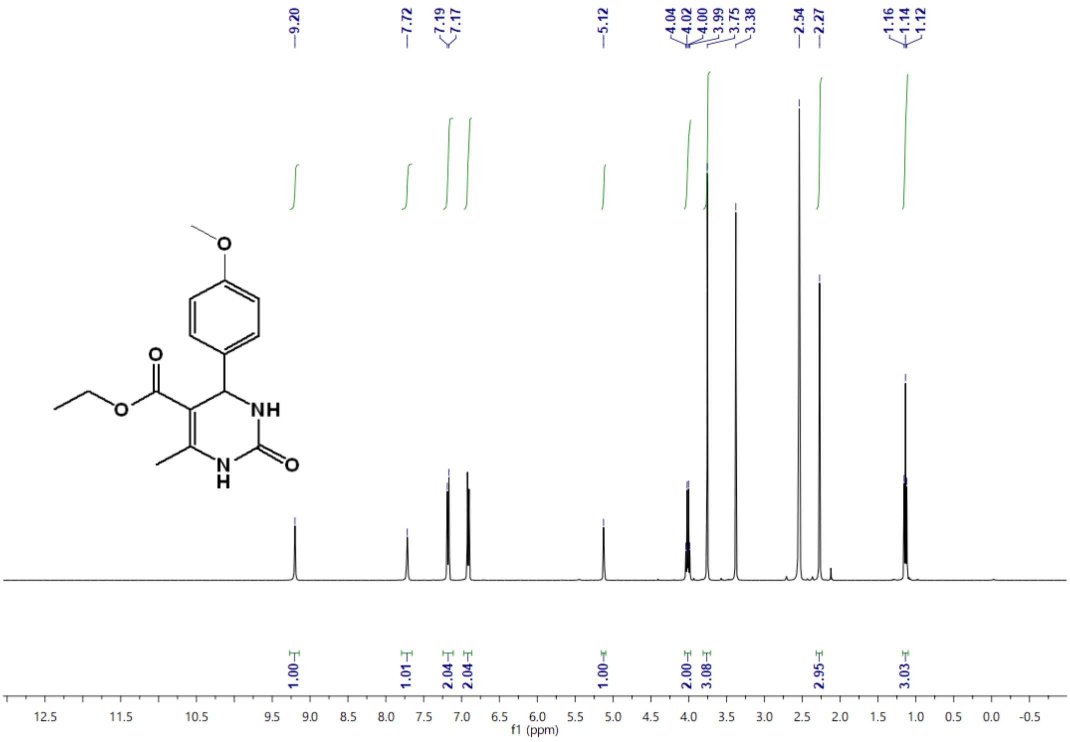

Figure S3 ^{1}H NMR **(400 MHz, DMSO-d$_6$)** spectrum of **4b**

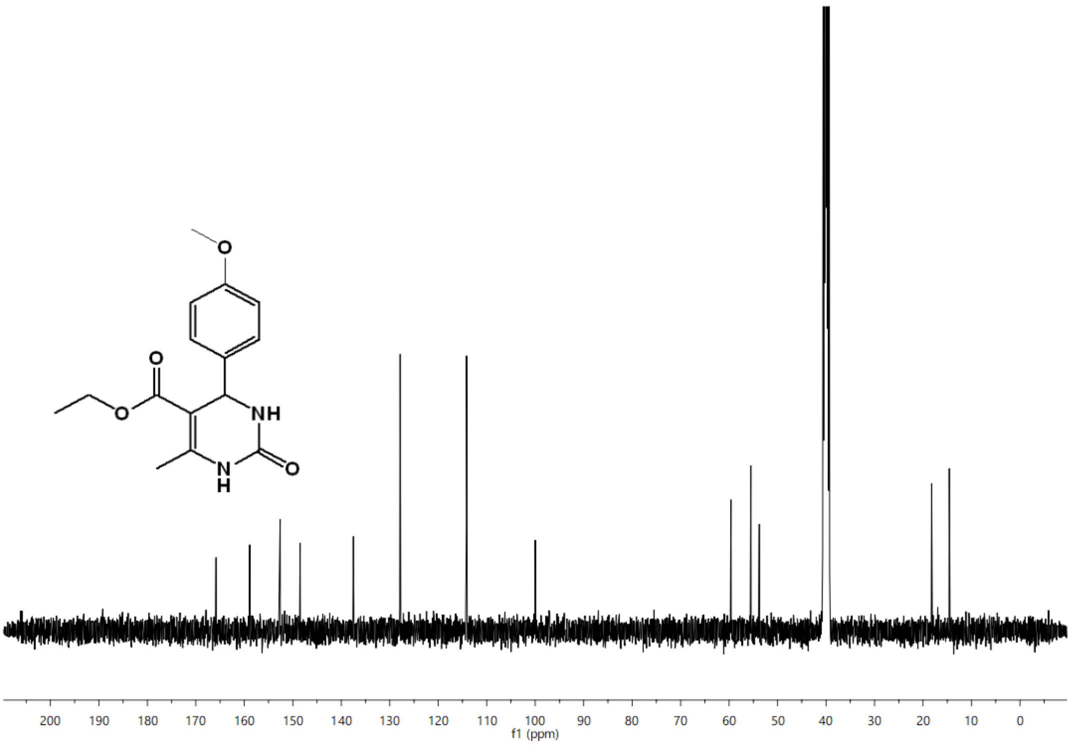

Figure S4 ^{13}C NMR **(101 MHz, DMSO-d$_6$)** spectrum of **4b**

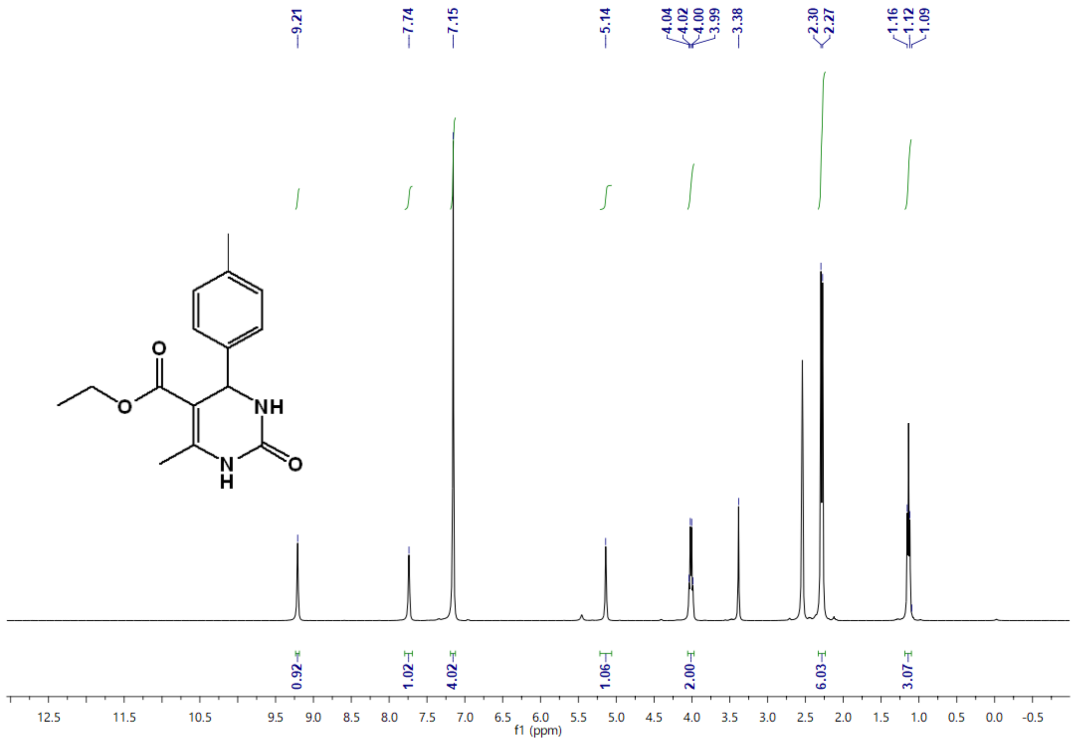

Figure S5 ^{1}H NMR **(400 MHz, DMSO-d6)** spectrum of **4c**

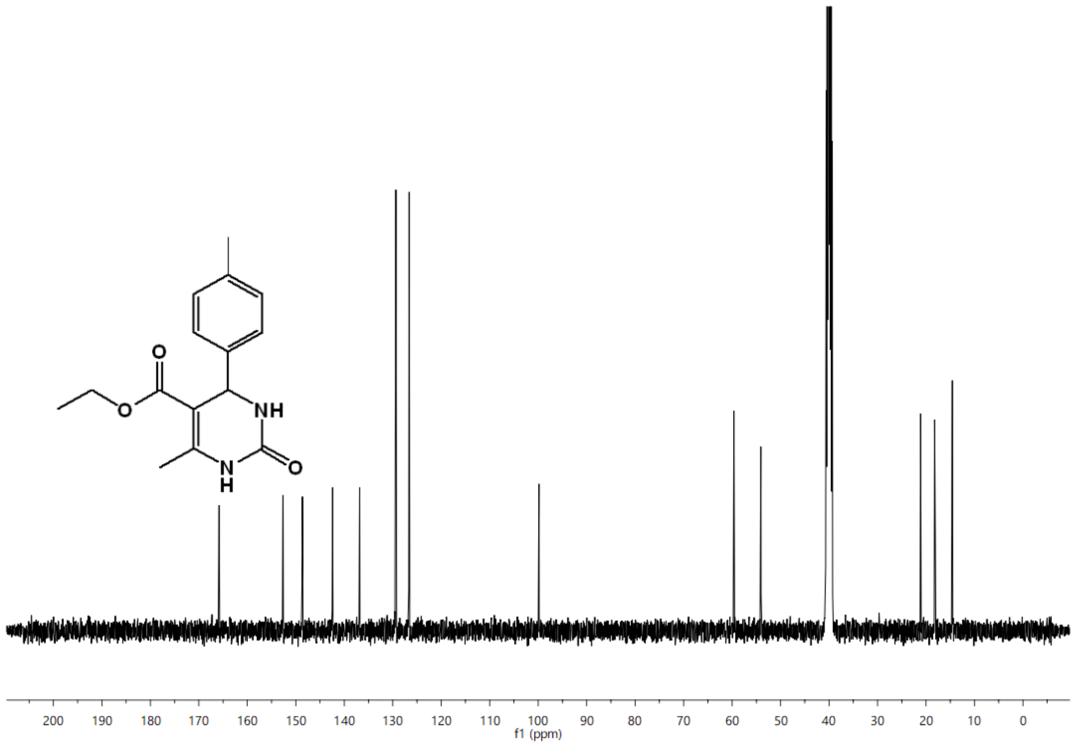

Figure S6 ^{13}C NMR **(101 MHz, DMSO-d6)** spectrum of **4c**

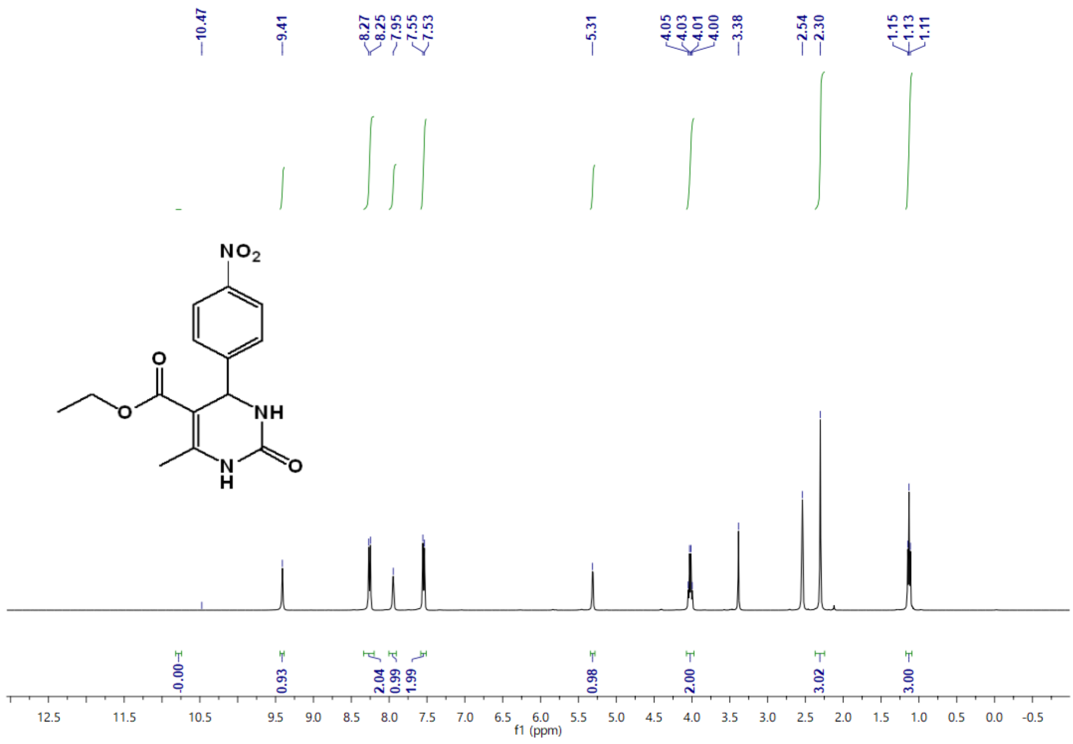

Figure S7 ^{1}H NMR **(400 MHz, DMSO-d$_6$)** spectrum of **4d**

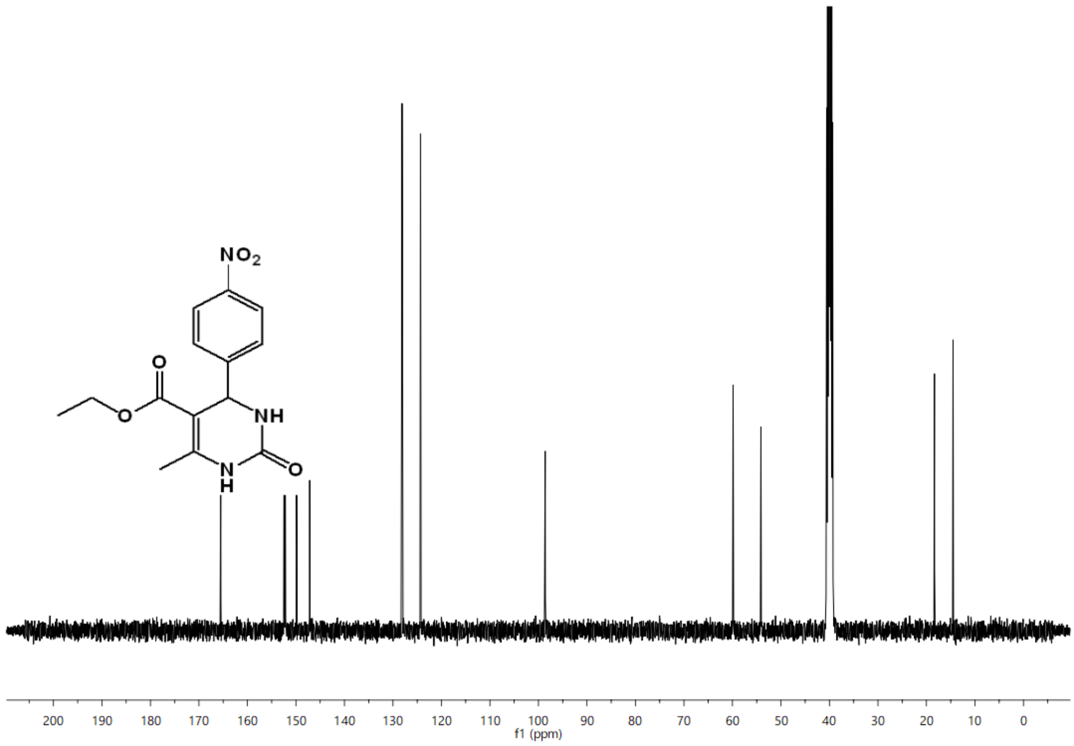

Figure S8 ^{13}C NMR **(101 MHz, DMSO-d$_6$)** spectrum of **4d**

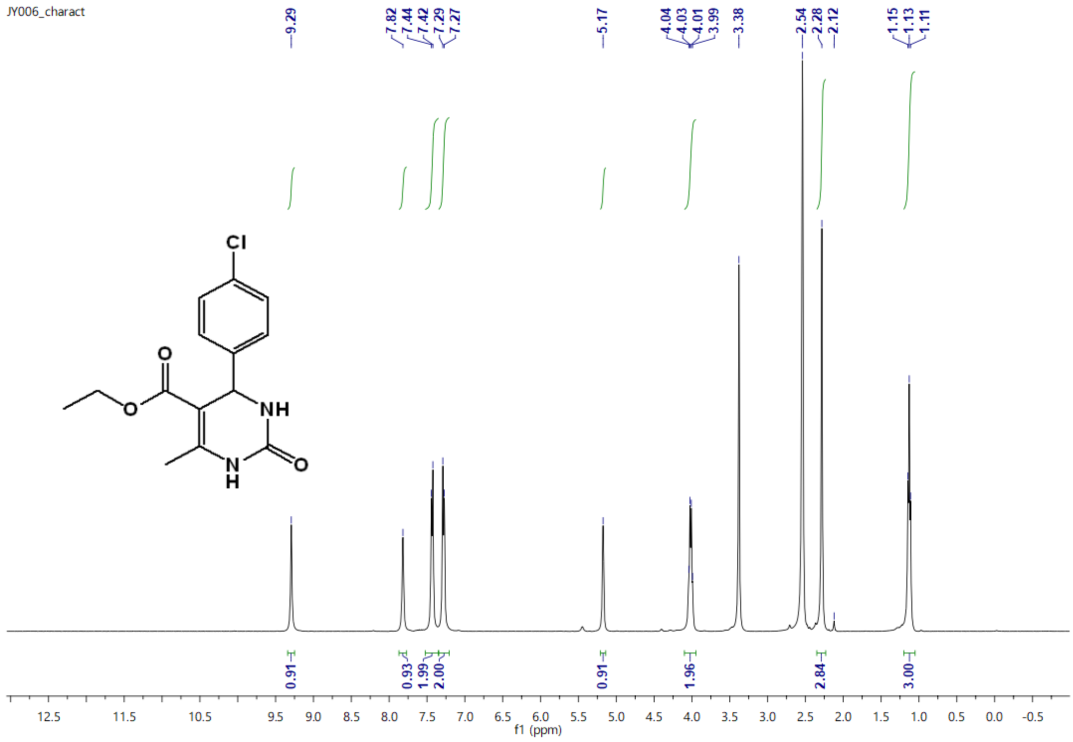

Figure S9 ^{1}H NMR **(400 MHz, DMSO-d$_6$)** spectrum of **4e**

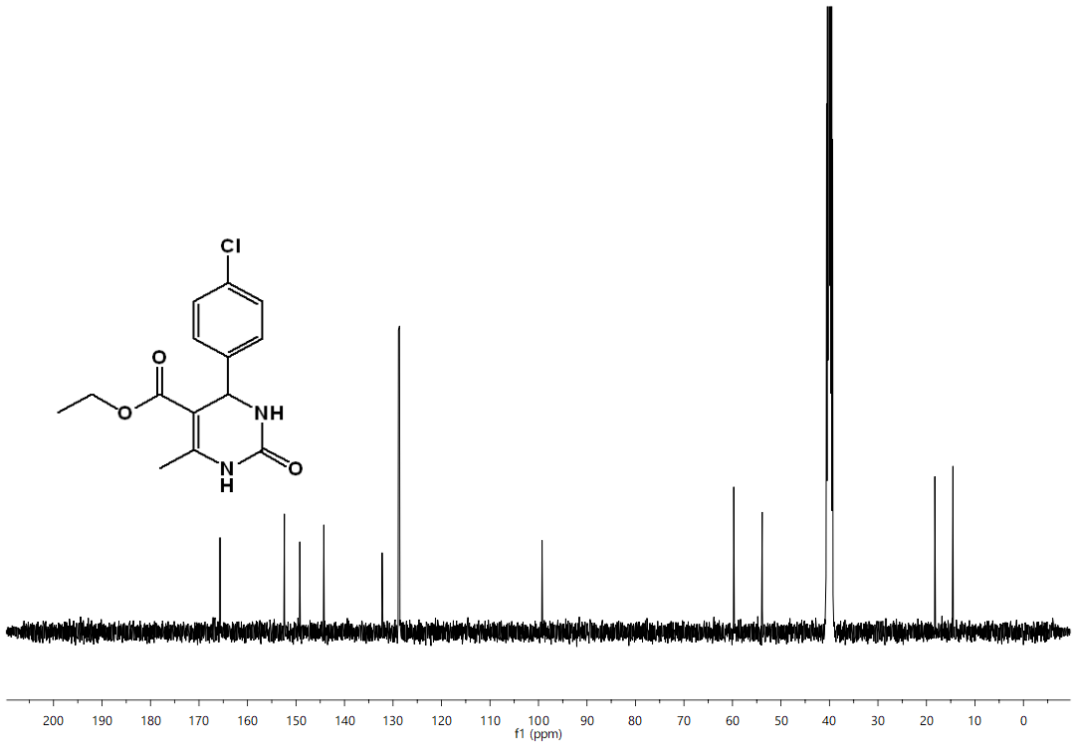

Figure S10 ^{13}C NMR **(101 MHz, DMSO-d$_6$)** spectrum of **4e**

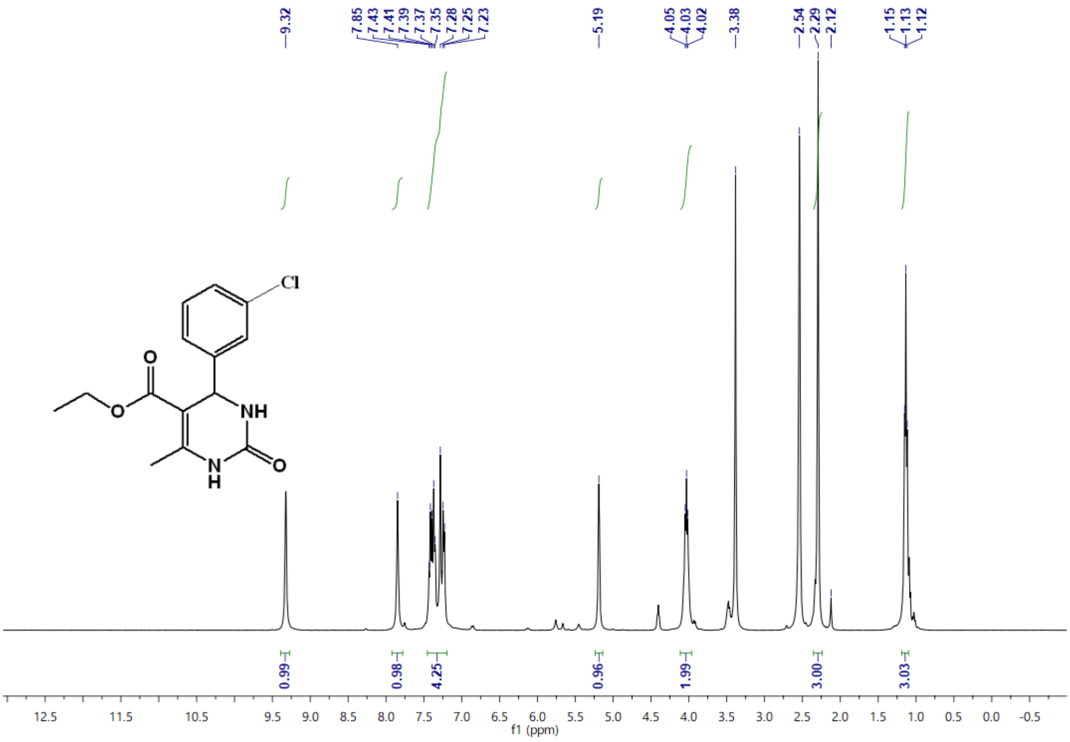

Figure S11 ^{1}H NMR **(400 MHz, DMSO-d6)** spectrum of **4f**

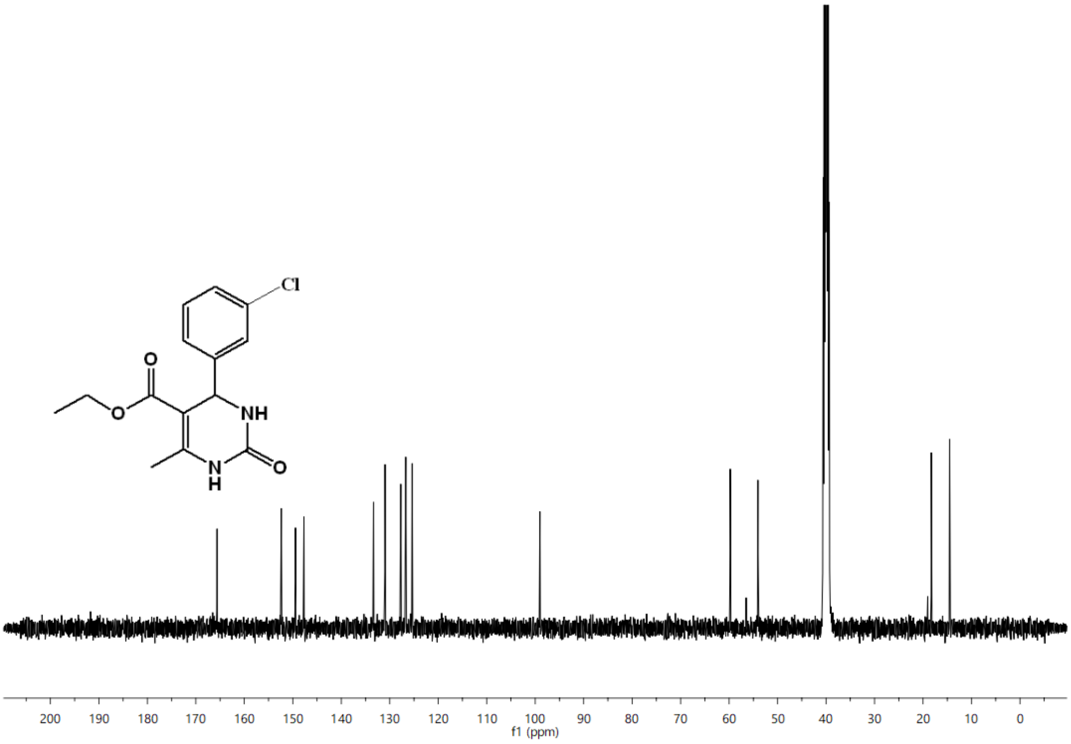

Figure S12 ^{13}C NMR **(101 MHz, DMSO-d6)** spectrum of **4f**

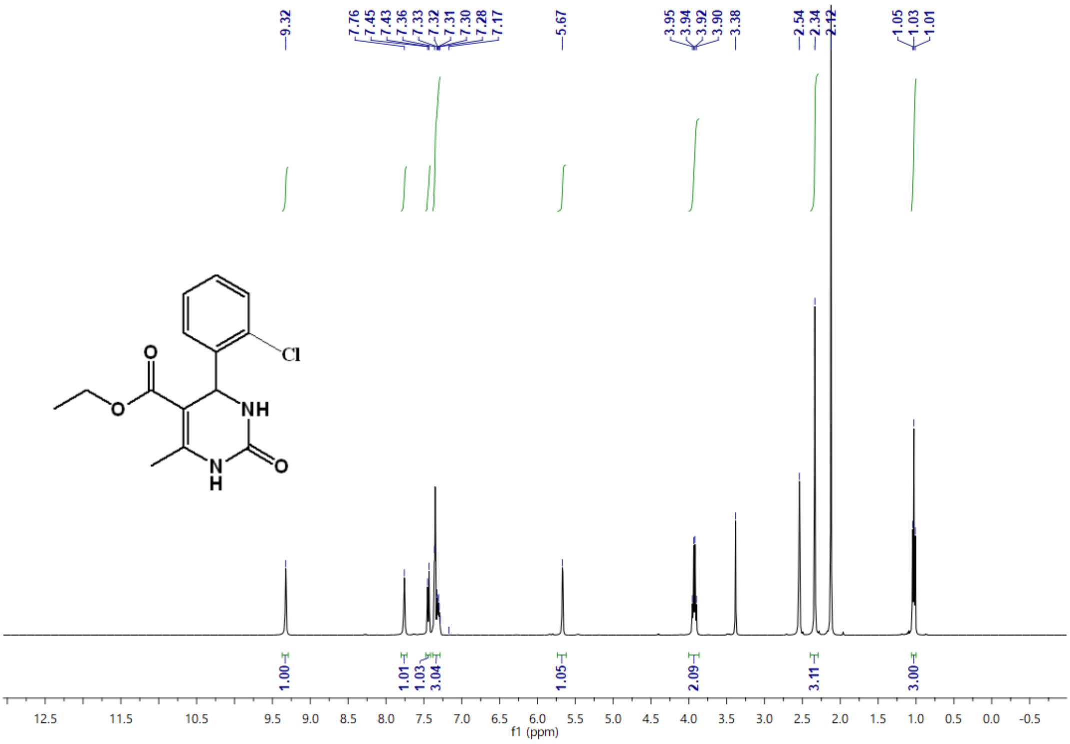

Figure S13 ^{1}H NMR **(400 MHz, DMSO-d6)** spectrum of **4g**

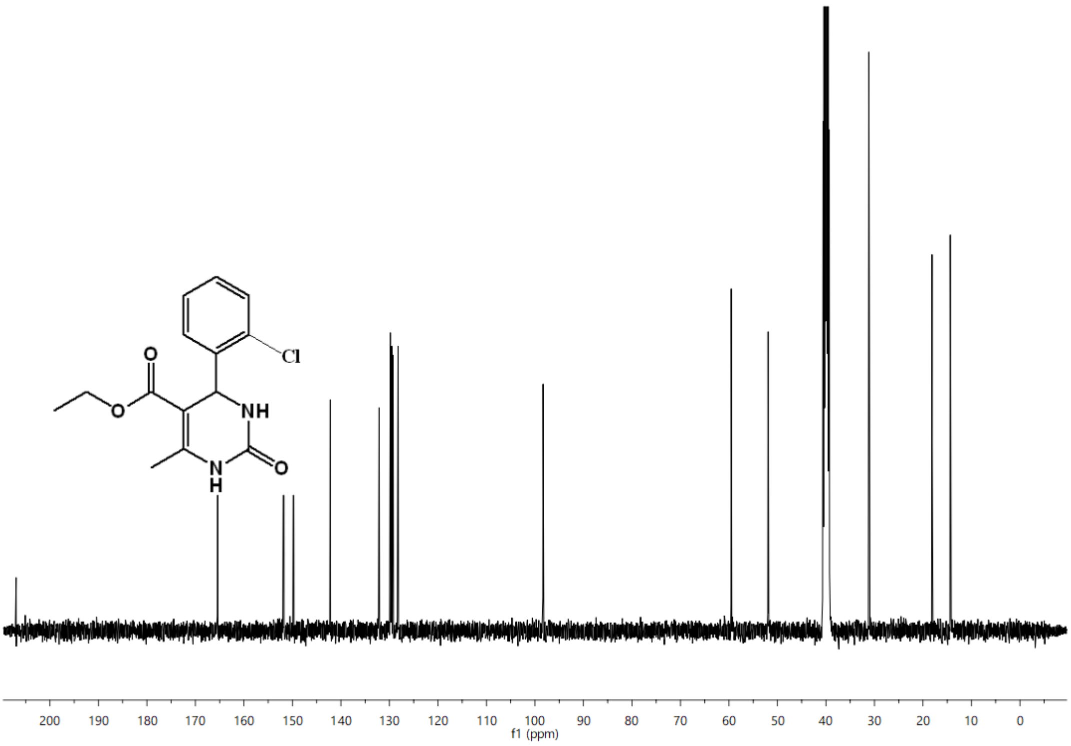

Figure S14 ^{13}C NMR **(101 MHz, DMSO-d6)** spectrum of **4g**

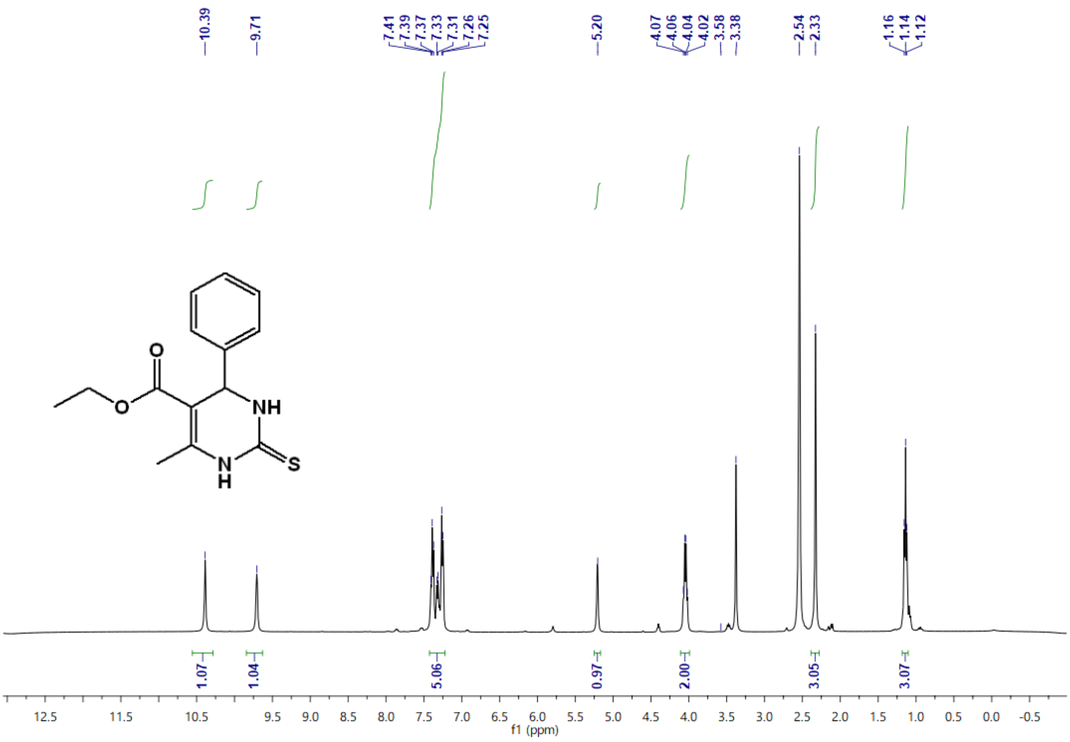

Figure S15 ^{1}H NMR **(400 MHz, DMSO-d$_6$)** spectrum of **4h**

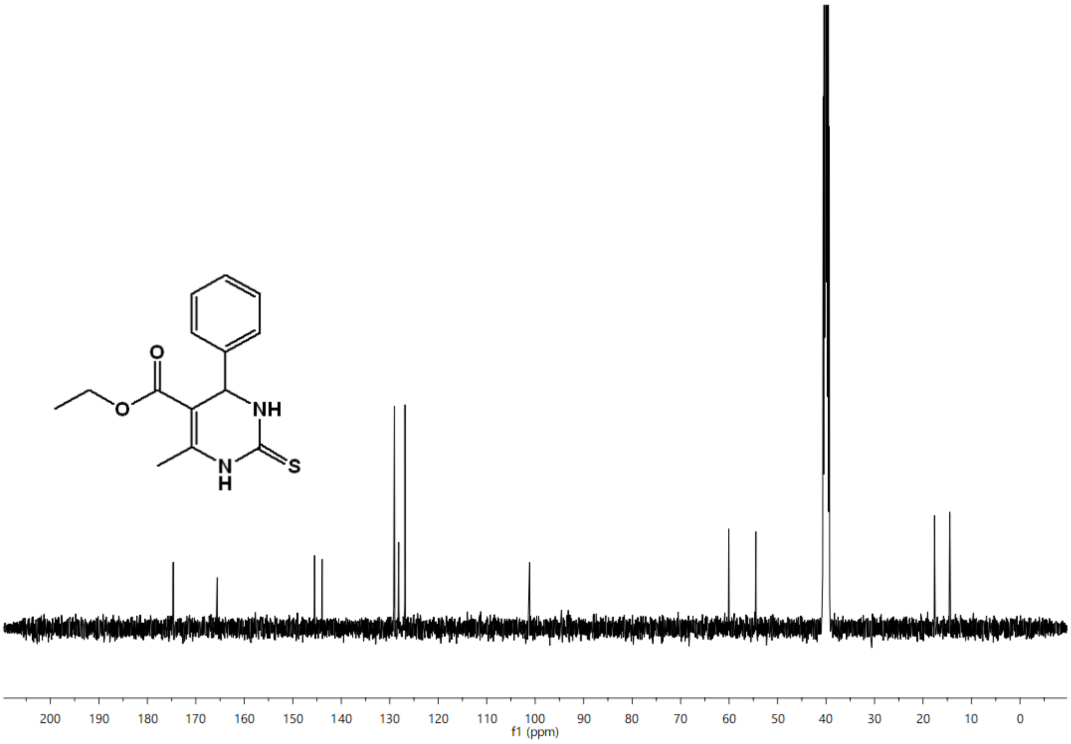

Figure S16 ^{13}C NMR **(101 MHz, DMSO-d$_6$)** spectrum of **4h**

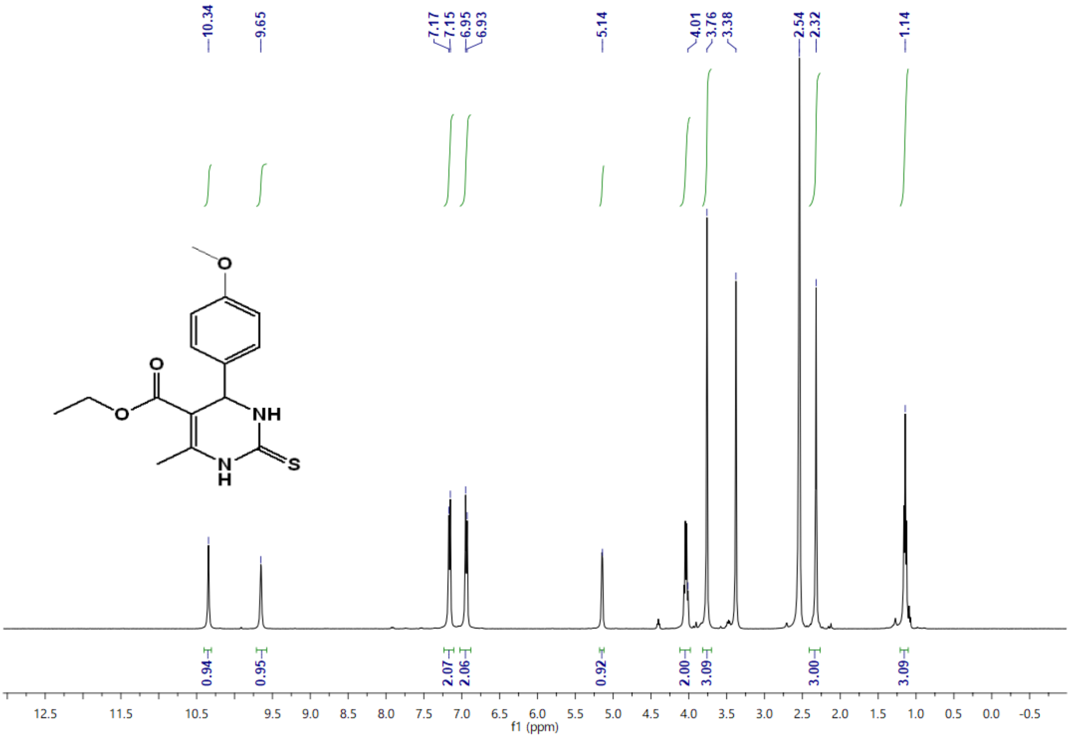

Figure S17 ^{1}H NMR **(400 MHz, DMSO-d6)** spectrum of **4i**

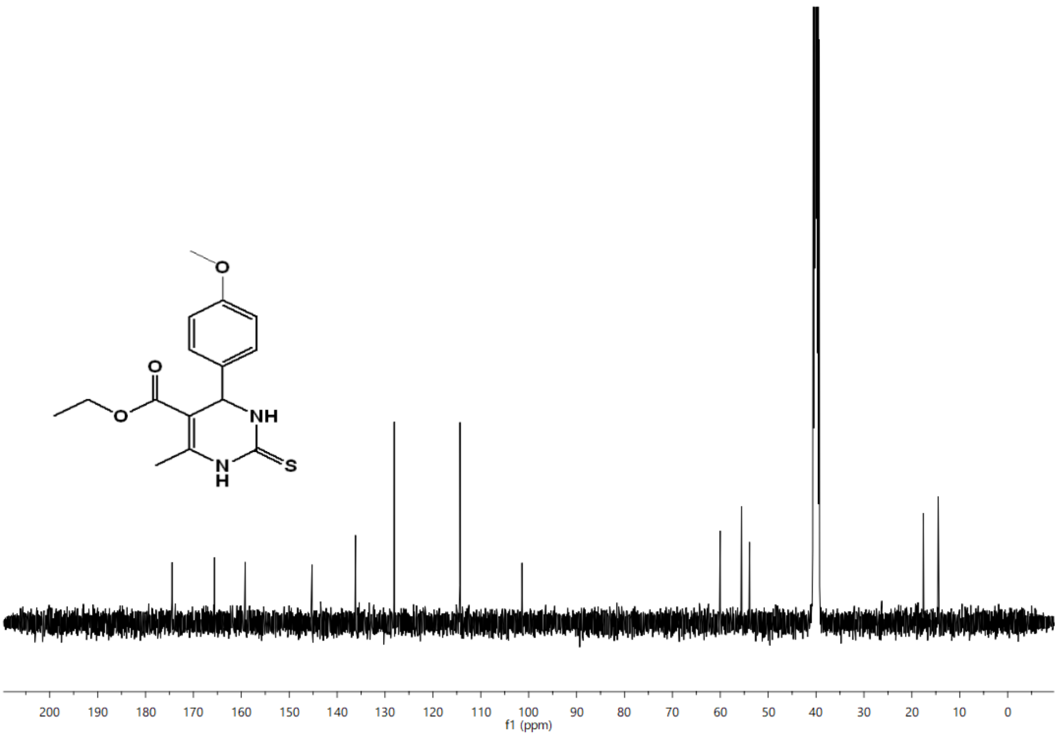

Figure S18 ^{13}C NMR **(101 MHz, DMSO-d6)** spectrum of **4i**

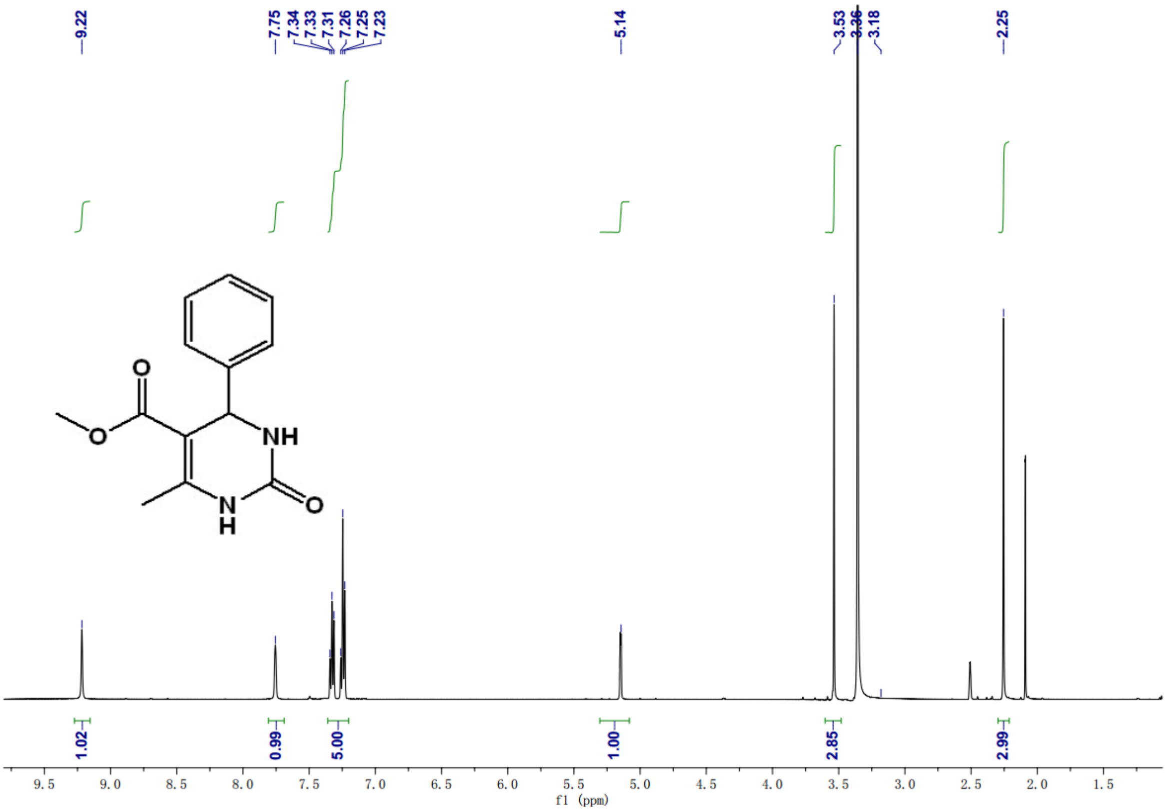

Figure S19 ^{1}H NMR **(400 MHz, DMSO-d**$_6$**)** spectrum of **4j**

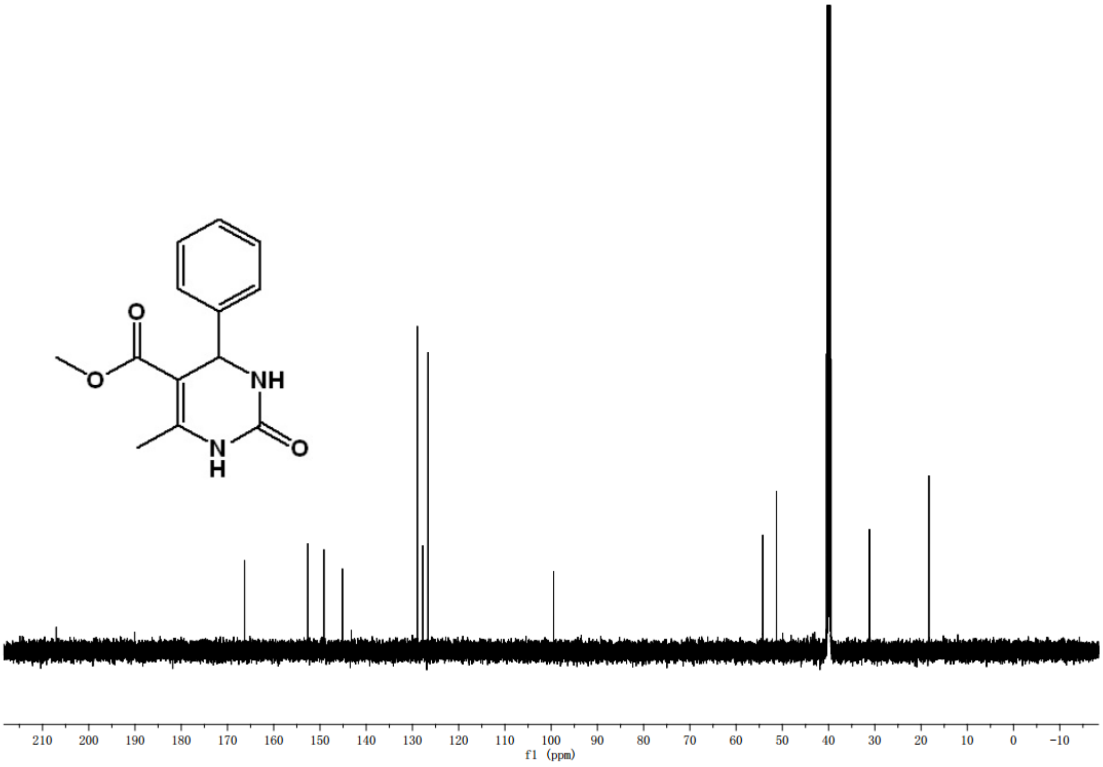

Figure S20 ^{13}C NMR **(101 MHz, DMSO-d**$_6$**)** spectrum of **4j**
